# Supplementary material for: Older Barbary macaques show limited capacity for self-regulation to avoid hazardous social interactions
Source: Commun Biol. 2022 Oct 12;5:1087. doi: 10.1038/s42003-022-04012-5 (PMC9556749; doi:10.1038/s42003-022-04012-5)
Supplement: Supplementary file 2 — Supplemental Material [file 42003_2022_4012_MOESM2_ESM.pdf]

Supplementary Material

Older Barbary macaques show limited capacity for self-regulation to avoid hazardous social interactions

Eva-Maria Rathke[1,2,3], Roger Mundry[1,2,3] & Julia Fischer[1,2,3]

[1]Cognitive Ethology Laboratory, German Primate Center, Leibniz Institute for Primate Research, Kellnerweg 4, 37077 Göttingen, Germany

[2]Department for Primate Cognition, Georg-August-University Göttingen, Göttingen, Germany

[3]Leibniz ScienceCampus Primate Cognition, Göttingen, Germany

Supplementary Methods

Supplementary Table 1. Study population by group, age, and sex.

Group PB (2017)				Group GB (2018)			
Female ID	Age [years]	Male ID	Age [years]	Female ID	Age [years]	Male ID	Age [years]
B121	<1	B122	<1	X93	4	C2	<1
B120	<1	A115	1	V71	5	C3	<1
Z104	2	A118	1	T63	6	B123	1
T60	5	Z102	2	R50	7	A111	2
T61	5	X95	3	R51	7	A113	2
R52	6	X87	3	R53	7	X82	4
O30	8	V72	4	O32	9	X92	4
O31	8	V73	4	M20	10	V70	5
M21	9	V74	4	M23	10	T61	6
L11	10	T60	5	M24	10	T62	6
K431	11	R52	6	K430	12	T63	6
H412	13	R55	6	K432	12	R57	7
G400	14	R56	6	I420	13	P41	8
D370	17	P40	7	I421	13	O31	9
C360	18	M21	9	I422	13	M23	10
B350	19	L10	10	H410	14	L14	11
B353	19	L12	10	F390	16	K430	12
B355	19	I421	12	E380	17	K431	12
A330	20	I422	12	B354	20	G401	15
A331	20	H410	13	Y310	23	G402	15
Z320	21	H412	13	Y311	23	D372	18
T268	26	H416	13	W291	25	B352	20
T274	26	G403	14	T261	27	T262	27
T277	26	F391	15	T264	27		
P227	28	E380	16	T275	27		
M189	30	D370	17	R248	28		
		C361	18	R253	28		
		A337	20	O200	30		
		A340	20				
		Z321	21				
		W283	24				
		U282	25				
		R247	27				
		O202	29				
		M189	30				

Note: Age is given for the respective years in which the data were collected.

Supplementary Table 2. Statistical software and R packages used, with version number and reference

Software/Package	Version	Reference
R	4.0.3	[1]
MASS	7.3.54	[2]
car	3.0-10	[3]
lme4	1.1-25	[4]
brms	2.14.4	[5,6]
lmerTest	3.1.3	[7]
irr	0.84.1	[8]
EloRating	0.43	[9]

Supplementary Table 3. Description of the different response types and sample size for the analysis of variation in response type to agonistic signals by females (model 4). Total N = 1594 responses.

Response	*N*	Description
Make room (MR)	303	The subject leans away from an approaching monkey.
Give ground (GG)	722	The subject walks away from an approaching monkey.
Present (PR)	47	The monkey approaches another monkey with its hindquarters first and presents its anogenital region towards the interaction partner. The subject may bend the front legs and/ or look back at the interaction partner.
Teeth chatter (TC)	522	The lips and corners of the mouth are retracted and the teeth and sometimes the gums are exposed. The mouth is opened and closed rapidly, sometimes with a flapping tongue. The teeth sometimes produce a clicking sound. The eyebrows and scalp are pulled up, and the ears are flattened against the head. Teeth chatter can occur in response to both affiliative and agonistic signals.

Supplementary Results

Supplementary Table 4. **Results of the full model for the probability of showing any response (model 3)**

Term	Est.	SE	Cl_{low}	Cl_{up}	c^2	*df*	*P*	min	max
Intercept	1.198	0.205	0.809	1.571			[1]	1.132	1.247
Receiver age [2]	0.194	0.214	-0.193	0.604				0.080	0.253
Signal Category [3]	0.005	0.232	-0.462	0.443				-0.052	0.124
Signaler age [4]	-0.314	0.084	-0.475	-0.155	13.22	1	<0.001	-0.347	-0.281
Signaler sex_male [5]	-0.233	0.210	-0.560	0.071	1.28	1	0.258	-0.365	-0.133
Receiver age: Signal Category	-0.104	0.234	-0.568	0.319	0.27	1	0.606	-0.174	0.046

Note: estimates with standard errors (SE), confidence limits (lower and upper CI), and range of estimates (min, max) obtained when excluding the levels of random effects one at a time). *N* = 3115 events with *N* = 50 receivers.

[1] not indicated because being of very limited interpretability

[2] *z*-transformed to a mean of zero and a standard deviation (*sd*) of one; mean and *sd* the of original age were 14.9 and 8.2 years, respectively

[3] dummy coded with affiliative being the reference level

[4] *z*-transformed to a mean of zero and a standard deviation (*sd*) of one; mean and *sd* the of original age were 14.5 and 6.0 years, respectively

[5] dummy coded with female being the reference level

Supplementary Table 5. Results of the model of the type of response after aggressive signals (model 4).

Term	Estimate	Est. Error	Lower CI	Upper CI
MR_Intercept	-1.345	0.189	-1.735	-0.993
PR_Intercept	-3.337	0.431	-4.309	-2.609
TC_Intercept	-0.143	0.166	-0.471	0.180
MR_z.Receiver_age	-0.221	0.119	-0.462	0.009
MR_z.Signaller_age	0.161	0.109	-0.052	0.375
MR_Signaller_sexmale	0.401	0.242	-0.071	0.890
PR_z.Receiver_age	-0.281	0.274	-0.874	0.196
PR_z.Signaller_age	0.021	0.246	-0.480	0.486
PR_Signaller_sexmale	-0.336	0.482	-1.306	0.601
TC_z.Receiver_age	0.227	0.136	-0.038	0.498
TC_z.Signaller_age	-0.169	0.093	-0.348	0.016
TC_Signaller_sexmale	-0.934	0.200	-1.334	-0.557

Note: Indicated are estimated effects and standard errors as well as limits of 95% credible intervals. Signaler and receiver age were *z*-transformed to a mean of zero and a standard deviation of one; mean and *sd* for the original variables were 13.8 and 5.5 (signaler age) and 15.1 and 8.4 years (receiver age); sex was dummy coded with female being the reference category.

Supplementary Figure 1. Effects of receiver age on the type of response.

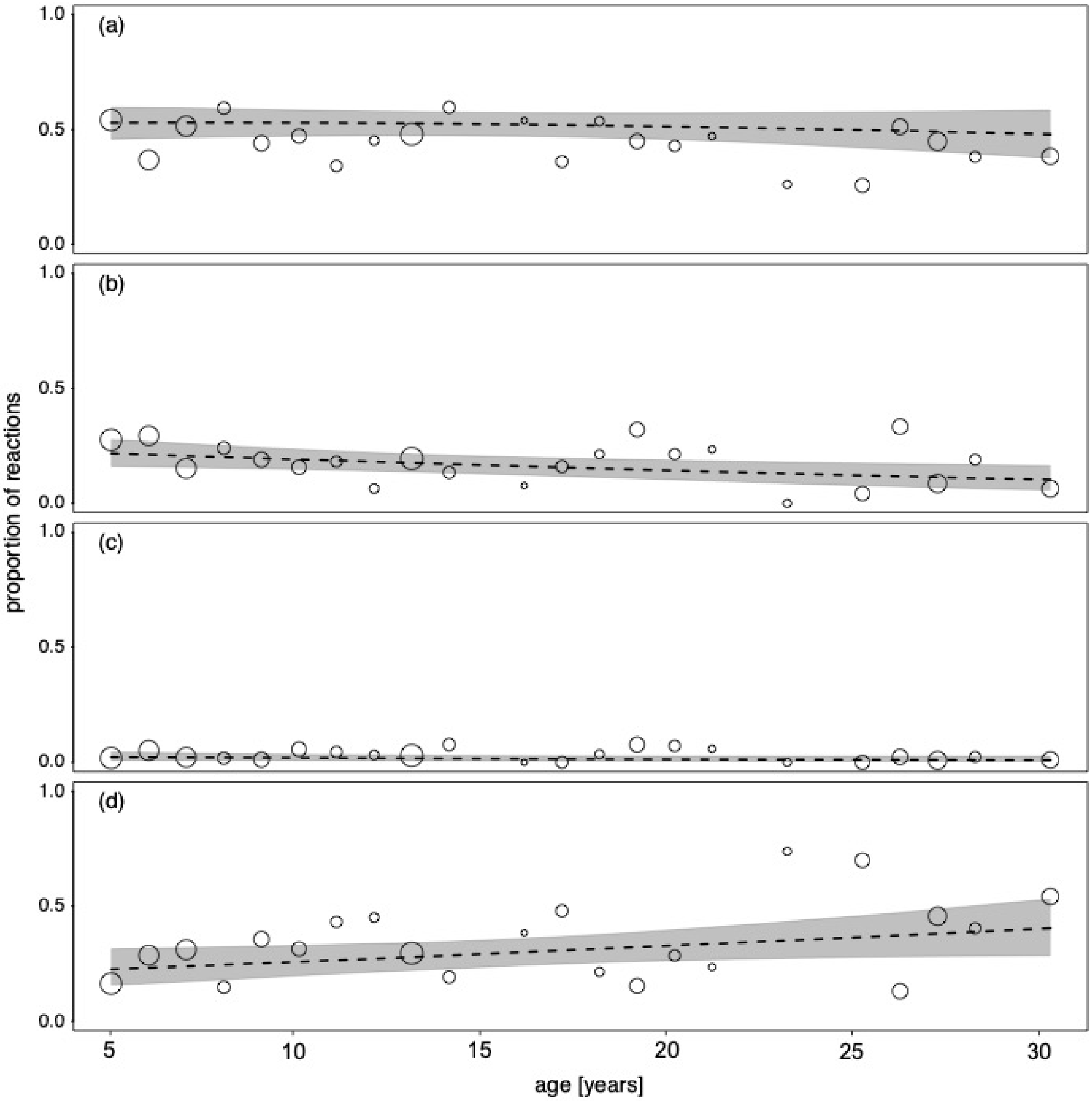

a Give Ground (N=722 events involving 50 receivers). **b** Make Room (N=303 events involving 46 receivers). **c** Present (N=47 events involving 29 receivers). **d** Teeth Chatter (N=522 involving 50 receivers). Dots show the occurrence probability of response type in a given year of age, whereby the area of the dots is proportionate to the total number of observed reactions (of all types) per year of age (range: 3 to 215). Dashed lines depict the fitted model (posterior median) and shaded polygons the limits of its 95% credible interval for both signaler age and sex centered to a mean of zero.

Supplementary Table 6. Results of the Likelihood ratio test for the analysis of the first look (model 5a).

Term	*df*	AIC	BIC	logLik	deviance	c^2	*df*	*P*
Null model	16	287.3	337.1	-127.7	255.3	-	-	-
Full model	18	289.8	345.8	-126.9	253.8	1.56	2	0.457

Note: N = 166 trials

Supplementary Table 7. Results of the model with duration of the first look as the response (model 5b).

Term	Est.	*SE*	*t*	*df*	Pr(>|t|)
(Intercept)	0.918	0.101	9.129	54.6	0.000
z.age [(1)]	-0.069	0.058	-1.189	135.5	0.237
type_n [(2)]	-0.077	0.076	-1.020	25.8	0.317
ID_sex_male [(3)]	0.218	0.119	1.827	23.7	0.080
picture_sexmale [(4)]	0.170	0.099	1.712	22.4	0.101
pair_second [(5)]	-0.171	0.093	-1.848	20.3	0.079
z.age:type_n	0.029	0.075	0.390	110.6	0.697

Note: Estimates, standard errors (*SE*), test statistic *t,* degrees of freedom (*df*), and *P*-value are given.

[(1)] z-transformed to a mean of zero and a standard deviation (*sd*) of 1
Mean and *sd* for the original variable were 13.89 and 7.75.

[(2)] dummy coded with agonistic facial expression being the reference level

[(3)] dummy coded with ID sex = female being the reference level

[(4)] dummy coded with picture ID sex = female being the reference level

[(5)] dummy coded with pair = first being the reference level

Supplementary Table 8. Results of the Likelihood ratio test for the analysis of the total looking time (model 5b)

Term	Df	AIC	BIC	logLik	deviance	c^2	*df*	*P*
Null model	16	435.5	485.3	-201.8	403.5	-	-	-
Full model	18	438.5	494.5	-201.3	402.5	1.02	2	0.601

Note: N = 166 trials.

Supplementary Table 9. Results of the model with total looking time as the response.

Term	Est.	*SE*	*t*	*df*	Pr(>|t|)
(Intercept)	1.523	0.149	10.254	89.972	0.000
z.age [1]	-0.082	0.112	-0.728	38.321	0.471
type_n [2]	-0.014	0.122	-0.117	39.142	0.907
ID_sex_male [3]	0.216	0.207	1.043	25.598	0.306
picture_sex_male [4]	0.140	0.112	1.248	51.259	0.218
pair_second [5]	-0.376	0.117	-3.229	56.980	0.002
z.age:typen	0.001	0.152	0.005	12.385	0.996

Note: Estimates (Est.), standard errors (*SE*), test statistic *t*, degrees of freedom (*df*), and *P* values for the predictor variables age, picture type, sex of the subject, sex of the animal shown in the picture and interaction between age and type are given.

[1] z-transformed to a mean of zero and a standard deviation (*sd*) of 1.
Mean and *sd* for the original variable were 13.89 and 7.75.

[2] dummy coded with agonistic facial expression being the reference level

[3] dummy coded with ID sex = female being the reference level

[4] dummy coded with picture ID sex = female being the reference level

[5] dummy coded with pair = first being the reference level

Supplementary Figure 2. Likelihood to manipulate the picture in relation to age.

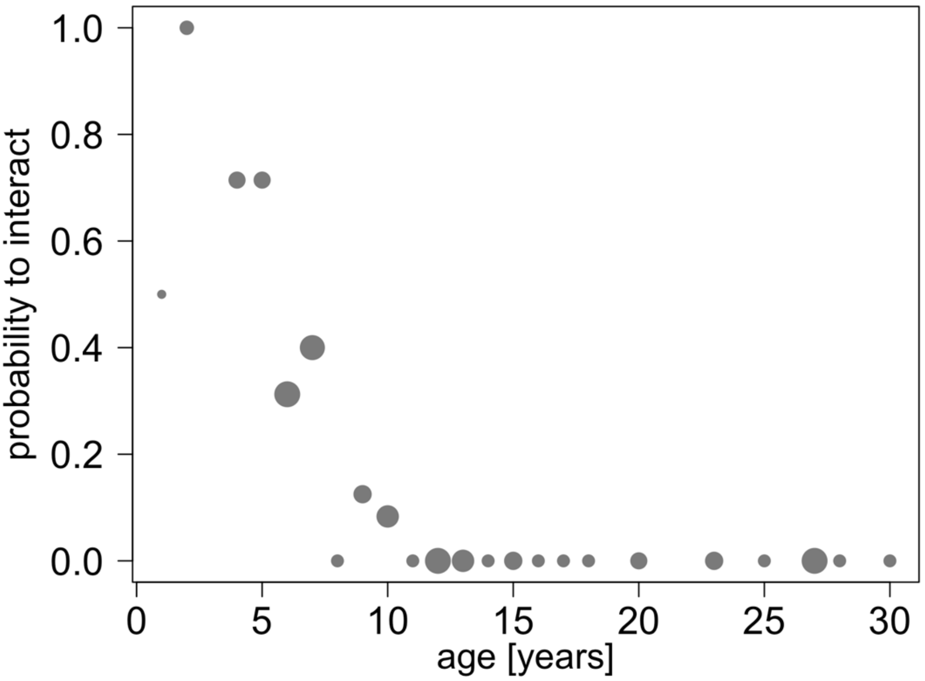

Proportion of occurrences that the subject touched or sniffed at the picture in relation to age.

Supplementary References

1. R Core Team. R: A Language and Environment for Statistical Computing. Preprint at https://www.r-project.org/ (2020).
2. Venables, W. N. & Ripley, B. D. *Modern Applied Statistics with S*. (Springer, 2002).
3. Fox, J. & Weisberg, S. *An R Companion to Applied Regression*. (Sage, 2019).
4. Bates, D. M. Fitting linear mixed models in R. Using the lme4 package. *R News* **5**, 27–30 (2005).
5. Bürkner, P.-C. Advanced Bayesian Multilevel Modeling with the R Package brms. *The R Journal* **10**, 395–411 (2018).
6. Bürkner, P.-C. **brms** : An *R* Package for Bayesian Multilevel Models Using *Stan*. *Journal of Statistical Software* **80**, 1–28 (2017).
7. Kuznetsova, A., Brockhoff, P. B. & Christensen, R. H. B. lmerTest Package: Tests in Linear Mixed Effects Models. *Journal of Statistical Software* **82**, 1–26 (2017).
8. Gamer, M., Lemon, J. & Fellows, I. P. S. irr: Various Coefficients of Interrater Reliability and Agreement. R package version 0.84.1. . Preprint at (2019).
9. Neumann, C. & Kulik, L. EloRating: Animal dominance hierarchies by Elo rating R. *R package version 0.43.* http://CRAN.R-project.org/package=EloRating Preprint at (2014).
