# Supplementary material for: Older Barbary macaques show limited capacity for self-regulation to avoid hazardous social interactions
Source: Commun Biol. 2022 Oct 12;5:1087. doi: 10.1038/s42003-022-04012-5 (PMC9556749; doi:10.1038/s42003-022-04012-5)
Supplement: Supplementary file 3 — Description of Additional Supplementary Files [file 42003_2022_4012_MOESM3_ESM.pdf]

Description of Additional Supplementary Files

File name: Supplementary Movie 1

Description: A juvenile male Barbary macaque attends to the presentation of the photograph of a conspecific's (neutral) facial expression.
